# The Attentional Window, Search Difficulty and Search Modes: A Reply to Commentaries on Theeuwes (2023)

**DOI:** 10.5334/joc.305

**Published:** 2023-07-06

**Authors:** Jan Theeuwes

**Affiliations:** 1 Vrije Universiteit Amsterdam, Amsterdam, The Netherlands

**Keywords:** attention capture, attentional suppression, visual search

## Abstract

With great pleasure I studied the commentaries of my esteemed colleagues to my opinion paper “*The Attentional Capture Debate: When Can We avoid Salient Distractors and When Not?*” (Theeuwes, 2023). I thought the comments were to-the-point and provocative and I believe that these kinds of exchanges will help the field to move forward in this debate. I discuss the most pressing concerns in separate sections where I have grouped commonly raised issues.

## 1. Parallel-serial search and the attentional window

Across the commentaries there is consensus that if the target is a salient singleton and stands out from the background, search can be performed in parallel across the display. In these circumstances, there is attentional capture as long as the distractor is more salient than the target. This the basic finding of Theeuwes (1991,1992) and represents the top left quadrant of Table 1 of Lien and Ruthruff’s (2023) commentary. It is less clear however, whether there is capture when the target is not a singleton and is therefore less salient. I have argued that in those circumstances, the target cannot be found by means of parallel search and therefore search needs to proceed in another fashion. I have suggested that when search for the target cannot be performed in parallel across the display, search needs to proceed in a clump-wise, serial fashion. In those circumstances, it is less likely that a salient distractor has the ability to capture attention. One possible mechanism that allows to find a non-salient target is to reduce the extent to which attention is spread across the display. The notion of a reduced attention window, that I first introduced in a paper published in 1994 (Theeuwes, 1994), is a way to describe the (partly) serial search that is needed to find a nonsalient target. In their commentary, Gaspelin et al (2023) reason that with such a small attentional window there cannot be any guidance by featural information anymore. While this was also claimed by the original feature integration theory (FIT) of Treisman and Gelade (1980), later findings indicated that feature information can guide the (serial) deployment of attention (Wolfe, 2021; Treisman & Sato, 1990). For example, we have shown that during serial search when the attentional window is small, participants are guided by featural information as they selectively can search among the relevant subset of elements (for example the red items) and completely ignore the irrelevant set (i.e., the green items) (Kaptein, Theeuwes, and Van der Heijden, 1995; see also Egeth, Virzi, & Garbart, 1984). Indeed, counter to what Gaspelin et al (2023) claim, in the target article I argue that during serial search “*there is massive guidance by the priority map”* (Theeuwes 2023, p. 5) and “*Indeed, search is guided by the display characteristics and even within a search episode during a trial, the attentional window can vary in size*” (Theeuwes, 2023, p.5). This latter claim fits very well with the notion discussed in Stoermer & Noonan’s (2023) commentary, in which they argue that search is better conceived as a dynamic process in which search can vary from parallel to serial within and between trials. Also, note that our view is different from Liesefeld and Müller (2023; see also Liesefeld, & Müller, 2020) who reason that when the attentional window is small, there is no featural guidance and clump-scanning is completely “*unguided and idiosyncratic*” (Liesefeld & Müller, 2023, p. 3).

## 2. Search difficulty

In the target article, I claimed that capture by salient items is not found when the target is non-salient (i.e., difficult to find) and search cannot be done by the first preattentive parallel stage of processing. The question is then when is search so difficult that it cannot be done by parallel processing? The issue of search difficulty is discussed in all commentaries. I argued that search is difficult when the target no longer pops-out from the background; a condition created by heterogenous displays that is assumed to induce the feature search mode. Table 1 of Lien and Ruthruff (2023) is insightful here as it makes both a distinction between easy (parallel) - difficult (serial) search and whether the target is a singleton or not. This distinction is related to the *feature search mode* in which the target is a nonsingleton versus the *singleton detection mode* when the target is a singleton. In our 2004 paper (Theeuwes, 2004) the target was nonsingleton but because display size was large (20 elements) the target stood out from its immediate background (i.e., it was locally unique) and could therefore be detected by means of parallel search. Because search was performed in parallel there was also attentional capture. Lien and Ruthruff (2023) argue that it is also possible to get capture when search is difficult especially when the singleton is an abrupt onset. Indeed, classic work of Yantis & Jonides (1984) already demonstrated that abrupt onset captures attention even when participants engage in serial search; however, in those very same circumstances, static singletons such as color singletons do not capture attention (Jonides & Yantis, 1988; see also Theeuwes 2023 for a discussion of these findings).

When discussing search difficulty, Liesefeld and Müller (2023) make a distinction between salience and discriminability, the latter term, according to Liesefeld and Müller, is assumed to represent “*the similarity of a psychical stimulus and a mental representation (the search template)*” (p.2). While both aspects play a role during search, we believe that salience is a critical prerequisite for (parallel) search (see for example, Wang & Theeuwes, 2020) while discriminability as defined by Liesefeld and Müller (2023) plays a role in post-selection processes. For example, if an item is selected for further processing, participants need to decide whether the selected object is indeed the target. The match with the search template becomes critical here. Similarly, Noonan and Stoermer (2023) discuss how knowledge regarding the target affects serial search, and it is likely that also this affects the speed with which one can decide that the selected object is the target one was looking for. I assume that saliency of the target determines whether search can be conducted in parallel or not, and discriminability determines the speed with which a participant can decide that the object selected is the target or not. This latter factor plays a crucial role in the speed with participants can disengage from a location after selecting an object presented at that location. Indeed, if the object selected does not look like the target at all (as for example in Folk et al., 1992 contingent capture paradigm) disengagement can be extremely fast (see Theeuwes et al., 2000; Born, Kerzel & Theeuwes, 2011).

## 3. Feature-search and singleton-detection modes

While I have questioned the existence of the implicitly assumed search modes (feature search versus singleton detection mode; cf. Bacon & Egeth, 1994) the research field has widely accepted the notion of search modes as a way to explain that during feature search, top-down control prevents attentional capture. Contrary to this view, I have argued that these search modes do not represent a top-down strategy but instead are induced by the display configurations (Theeuwes, 2010; 2023). Indeed, when the target does not pop-out from the display, one needs to use what has been labelled feature search mode.

While the existence of search modes is widely accepted, it should be noted that the evidence for these modes is relatively scarce. In their commentaries Liesefeld and Müller (2023) and Gaspelin and Luck (2023) point to the study of Leber and Egeth (2006) and argue that this study provides “*the most convincing proof of the existence of search modes*” (Liesefeld and Müller, 2023, p. 3). While this study indeed shows that during feature search, there was no attentional capture while at the same time, search was still conducted in parallel (no display size effect), it is important to note that Leber and Egeth (2006) were only able to induce such a “top-down” feature search mode after extensive training involving at least 480 feature search trials. Indeed, with a lot of practice, search may become very efficient (Theeuwes et al, 2022), allowing relatively fast serial search in which disengagement becomes very fast. As such one can question how convincing this “proof” of this top-down-induced search mode really is.

## 4. Evidence for attentional capture by salient distractors

I have argued before (Theeuwes, 2010, 2019; Theeuwes et al., 2000) that it is possible that salient singletons capture attention, but that attention may be disengaged quickly especially when the distractor does not resemble the target (the disengagement hypothesis; see Theeuwes et al., 2000). In those circumstances, it may seem that participants simply ignore the salient singleton which is then typically interpreted as evidence that top-down control prevented attentional capture. In their commentaries, both Gaspelin et al., (2023) as well as Lien and Ruthruff (2023) discuss several tasks that resemble the classic contingent capture paradigm of Folk et al., (1992). Typically, in these tasks matching cues capture attention and nonmatching cues are ignored (see for example, Figure 2 of Lien and Ruthruff).

Gaspelin et al. (2023) argue that these findings are problematic for my account and cannot be explained by “rapid disengagement from the cue” as according to Gaspelin et al. “there is now strong evidence against this viewpoint” (p. 3 footnote). While according to the commentaries the evidence against my rapid disengagement hypothesis is strong, a recently published paper by Klink et al., (2023) provides the most clear and convincing evidence in favor of my rapid disengagement hypothesis thus far. Klink et al. (2023) measured neuronal activity of V4 neurons of macaques performing an eye movement version of the additional singleton paradigm. While based on eye-movements alone, it appeared that the distractor was successfully inhibited, neuronal activity of V4 neurons showed that there was an initial epoch of salient distractor enhancement, which was followed by suppression, later than 150 ms (see Figure 3B of Klink et al., 2023). This clearly shows that initial attentional capture was followed by rapid attentional disengagement. Crucially, the initial capture by the salient pop-out distractor never went away, even though the monkeys were highly overtrained (monkey 1 performed 34,543 trials and monkey 2 performed 13,815 trials). It is important to note that these findings also show that even when there is no measurable effect of a non-matching cue on reaction time (as in contingent capture tasks) this does not necessarily mean that the cue did not capture attention. In several previous papers, I have made this very same argument to explain the absence of attentional capture for a non-matching cue in contingent capture paradigms. There is capture by the salient non-matching cue but with a delay between cue and search display (which is typically the case in these kind of paradigms), this effect does not show up in RT (Theeuwes, 2010; Theeuwes et al., 2000)

In their commentary, Gaspelin et al. (2023) discuss several eye movement studies (e.g., Gaspelin et al., 2017) showing that the first saccade goes to the target and never to the salient distractor (Figure 1 of Gaspelin et al., 2023 commentary). On the basis of these findings Gaspelin et al (2023) conclude that *”the salient singleton did not capture attention and was actually suppressed: It was fixated on only 5% of trial when present”*. These findings are fully consistent with Klink et al (2023): the monkeys never fixated the location of the salient color distractor and the eyes went straight to the target singleton. Yet, while the eyes went straight to the target, Klink et al. showed that there was initial attentional enhancement at the location of the color distractor, indicating that the conclusion of Gaspelin et al. that there was no attentional capture is not correct. As such, the method of measuring eye movements to provide evidence that attention never went to a salient object is arguably flawed.

Finally, the findings of Klink et al (2023) are fully consistent with the stimulus driven account that I have described before (Theeuwes, 2010, 2018, 2019). According to this account suppression can only take place after attention has been captured (even for the briefest moment) by the salient distractor. This account is unlike the signal suppression account of Gaspelin and Luck (2018) which claims that there can be top-down suppression of a salient distractor without the need for spatial attention to be directed to that location. The results of Klink et al demonstrate that before there is suppression, there is always initial capture even though this capture may not always be detectable by behavioral and eye movement measures.
